# *N*-(2,4,6-Trimethyl­phen­yl)-1,3-thia­zol-2-amine

**DOI:** 10.1107/S1600536812031315

**Published:** 2012-07-14

**Authors:** Ayesha Babar, Munawar Ali Munawar, M. Nawaz Tahir, Ather Farooq Khan, Muhammad Ilyas Tariq

**Affiliations:** a Institute of Chemistry, University of the Punjab, Lahore 54590, Pakistan; b Department of Physics, University of Sargodha, Sargodha, Pakistan; c Interdisciplinary Research Centre in Biomedical Materials, COMSATS Institute of Information Technology, Defence Road, Off Raiwind Road, Lahore, Pakistan; d Department of Chemistry, University of Sargodha, Sargodha, Pakistan

## Abstract

In the title compound, C_12_H_14_N_2_S, the dihedral angle between the 1,3,5-trimethyl­benzene and 1,3-thia­zol-2-amine groups is 73.15 (4)°. In the crystal, inversion dimers linked by pairs of N—H⋯N hydrogen bonds generate *R*_2_^2^(8) loops.

## Related literature

For background to the biological activities of thia­zoles, see: Wilson *et al.* (2001 ▶). For a related crystal structure, see: Caranoni & Capella (1982 ▶).
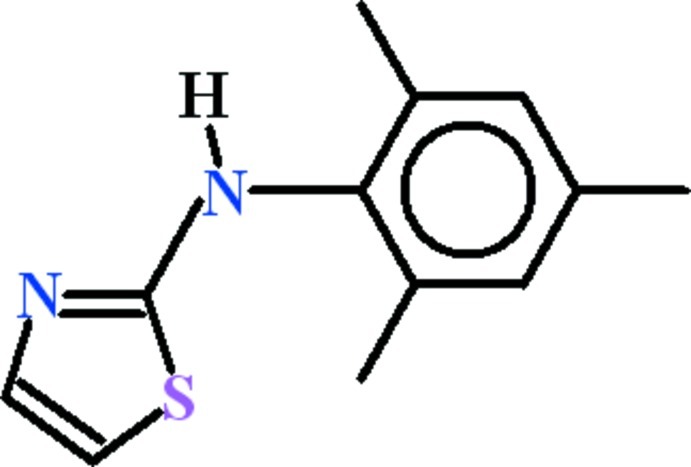


## Experimental

### 

#### Crystal data

C_12_H_14_N_2_S
*M* *_r_* = 218.31
Monoclinic, 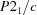

*a* = 14.2766 (6) Å
*b* = 7.0676 (2) Å
*c* = 13.8598 (6) Å
β = 118.736 (2)°
*V* = 1226.24 (9) Å^3^
*Z* = 4
Mo *K*α radiation
μ = 0.23 mm^−1^
*T* = 296 K
0.32 × 0.22 × 0.18 mm

#### Data collection

Bruker Kappa APEXII CCD diffractometer
Absorption correction: multi-scan (*SADABS*; Bruker, 2005 ▶) *T*_min_ = 0.929, *T*_max_ = 0.959
10086 measured reflections
2717 independent reflections
2196 reflections with *I* > 2σ(*I*)
*R*_int_ = 0.027

#### Refinement

*R*[*F* ^2^ > 2σ(*F* ^2^)] = 0.039
*wR*(*F* ^2^) = 0.116
*S* = 1.05
2717 reflections
140 parameters
H-atom parameters constrained
Δρ_max_ = 0.30 e Å^−3^
Δρ_min_ = −0.24 e Å^−3^

### 

Data collection: *APEX2* (Bruker, 2009 ▶); cell refinement: *SAINT* (Bruker, 2009 ▶); data reduction: *SAINT*; program(s) used to solve structure: *SHELXS97* (Sheldrick, 2008 ▶); program(s) used to refine structure: *SHELXL97* (Sheldrick, 2008 ▶); molecular graphics: *ORTEP-3 for Windows* (Farrugia, 1997 ▶) and *PLATON* (Spek, 2009 ▶); software used to prepare material for publication: *WinGX* (Farrugia, 1999 ▶) and *PLATON*.

## Supplementary Material

Crystal structure: contains datablock(s) global, I. DOI: 10.1107/S1600536812031315/hb6896sup1.cif

Structure factors: contains datablock(s) I. DOI: 10.1107/S1600536812031315/hb6896Isup2.hkl

Supplementary material file. DOI: 10.1107/S1600536812031315/hb6896Isup3.cml

Additional supplementary materials:  crystallographic information; 3D view; checkCIF report

## Figures and Tables

**Table 1:** Hydrogen-bond geometry (Å, °)

*D*—H⋯*A*	*D*—H	H⋯*A*	*D*⋯*A*	*D*—H⋯*A*
N1—H1⋯N2^i^	0.86	2.16	2.944 (2)	151

Symmetry code: (i) 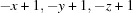.

**Fig. 1.**
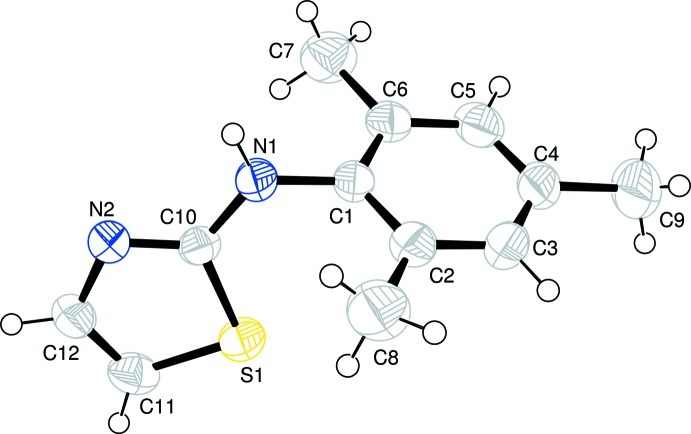
View of the title compound with displacement ellipsoids drawn at the 50% probability level.

**Fig. 2.**
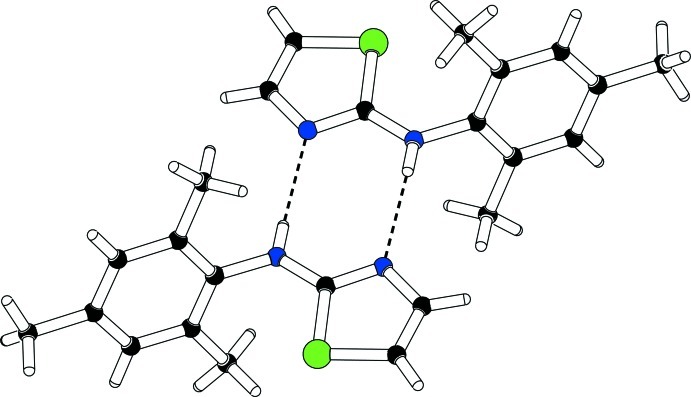
The partial packing (PLATON; Spek, 2009) which shows that molecules form dimers with R22(8) loops.

## supplementary crystallographic information

### Comment

Thiazole and its derivatives exhibit a large number of biological properties, for example antifungal and antibacterial (Wilson *et al.*, 2001) activities. As part of our studies in this area, the title compound (I, Fig. 1) has been synthesized and its crystal structure is now reported.

The crystal structures of 1,3-thiazol-2-amine (Caranoni & Capella, 1982) has been published which is related to (I), (Fig. 1).

In (I), the 1,3,5-trimethylbenzene moiety A (C1–C9) and 1,3-thiazol-2-amine group B (N1/C10/S1/C11/C12/N2) are planar with r.m.s. deviation of 0.0345 Å and 0.0031 Å, respectively. The dihedral angle between A/B is 73.15 (4)°. The molecules are linked into dimers due to H-bondings of N—H···N type with *R*_2_^2^(8) (Table 1, Fig. 2) ring motif.

### Experimental

A mixture of *N*-mesitylthiourea (1 equiv, 1.00 g, 4.58 mmol), 2-chloro-1,1-dimethoxyethane (1.5 equiv, 1.04 g, 6.8 mmol) and few drops of concentrated HCl were dissolved in water and methanol mixture (1:1) (100 ml). The reaction mixture was refluxed for 6 h. The reaction mixture was diluted with water (100 ml) and basified to pH 8 with aqeous NaOH. The resulting precipitate was filtered, washed with cold water and recrystallized from chloroform and hexane (3:1) solution as yellow prisms.

### Refinement

The H-atoms were positioned geometrically (C—H = 0.93–0.96 Å, N—H = 0.86 Å) and refined as riding with *U*_iso_(H) = *xU*_eq_(C, N), where *x* = 1.5 for methyl groups and *x* = 1.2 for other H atoms.

### Crystal data

**Table:**

C_12_H_14_N_2_S	*F*(000) = 464
*M**_r_* = 218.31	*D*_x_ = 1.183 Mg m^−^^3^
Monoclinic, *P*2_1_/*c*	Mo *K*α radiation, λ = 0.71073 Å
Hall symbol: -P 2ybc	Cell parameters from 2196 reflections
*a* = 14.2766 (6) Å	θ = 1.6–27.3°
*b* = 7.0676 (2) Å	µ = 0.23 mm^−^^1^
*c* = 13.8598 (6) Å	*T* = 296 K
β = 118.736 (2)°	Prism, yellow
*V* = 1226.24 (9) Å^3^	0.32 × 0.22 × 0.18 mm
*Z* = 4	

### Data collection

**Table:**

Bruker Kappa APEXII CCD diffractometer	2717 independent reflections
Radiation source: fine-focus sealed tube	2196 reflections with *I* > 2σ(*I*)
Graphite monochromator	*R*_int_ = 0.027
Detector resolution: 7.80 pixels mm^-1^	θ_max_ = 27.3°, θ_min_ = 1.6°
ω scans	*h* = −18→18
Absorption correction: multi-scan (*SADABS*; Bruker, 2005)	*k* = −8→9
*T*_min_ = 0.929, *T*_max_ = 0.959	*l* = −17→17
10086 measured reflections	

### Refinement

**Table:**

Refinement on *F*^2^	Secondary atom site location: difference Fourier map
Least-squares matrix: full	Hydrogen site location: inferred from neighbouring sites
*R*[*F*^2^ > 2σ(*F*^2^)] = 0.039	H-atom parameters constrained
*wR*(*F*^2^) = 0.116	*w* = 1/[σ^2^(*F*_o_^2^) + (0.0574*P*)^2^ + 0.3377*P*] where *P* = (*F*_o_^2^ + 2*F*_c_^2^)/3
*S* = 1.05	(Δ/σ)_max_ < 0.001
2717 reflections	Δρ_max_ = 0.30 e Å^−^^3^
140 parameters	Δρ_min_ = −0.24 e Å^−^^3^
0 restraints	Extinction correction: *SHELXL97* (Sheldrick, 2008), Fc^*^=kFc[1+0.001xFc^2^λ^3^/sin(2θ)]^-1/4^
Primary atom site location: structure-invariant direct methods	Extinction coefficient: 0.049 (4)

### Special details

**Table:**

Geometry. Bond distances, angles *etc*. have been calculated using the rounded fractional coordinates. All su's are estimated from the variances of the (full) variance-covariance matrix. The cell e.s.d.'s are taken into account in the estimation of distances, angles and torsion angles
Refinement. Refinement of *F*^2^ against ALL reflections. The weighted *R*-factor *wR* and goodness of fit *S* are based on *F*^2^, conventional *R*-factors *R* are based on *F*, with *F* set to zero for negative *F*^2^. The threshold expression of *F*^2^ > σ(*F*^2^) is used only for calculating *R*-factors(gt) *etc*. and is not relevant to the choice of reflections for refinement. *R*-factors based on *F*^2^ are statistically about twice as large as those based on *F*, and *R*- factors based on ALL data will be even larger.

### Fractional atomic coordinates and isotropic or equivalent isotropic displacement parameters (Å^2^)

**Table:**

	*x*	*y*	*z*	*U*_iso_*/*U*_eq_	
S1	0.28549 (3)	0.60626 (7)	0.20694 (3)	0.0510 (2)	
N1	0.37642 (11)	0.6678 (2)	0.42680 (11)	0.0489 (4)	
N2	0.46689 (11)	0.4830 (2)	0.35713 (11)	0.0457 (4)	
C1	0.28129 (12)	0.7598 (2)	0.41025 (12)	0.0417 (5)	
C2	0.25718 (15)	0.9401 (3)	0.36248 (14)	0.0503 (5)	
C3	0.16150 (17)	1.0218 (3)	0.34372 (15)	0.0619 (7)	
C4	0.09327 (16)	0.9380 (3)	0.37534 (15)	0.0640 (7)	
C5	0.12224 (14)	0.7638 (3)	0.42681 (14)	0.0579 (6)	
C6	0.21467 (13)	0.6720 (2)	0.44421 (12)	0.0464 (5)	
C7	0.24324 (19)	0.4809 (3)	0.49949 (19)	0.0689 (8)	
C8	0.3330 (2)	1.0470 (3)	0.3358 (2)	0.0774 (9)	
C9	−0.0095 (2)	1.0332 (5)	0.3547 (2)	0.1073 (13)	
C10	0.38520 (12)	0.5850 (2)	0.34340 (12)	0.0385 (4)	
C11	0.36100 (15)	0.4680 (3)	0.16835 (14)	0.0522 (6)	
C12	0.45154 (15)	0.4168 (3)	0.25688 (14)	0.0499 (6)	
H1	0.43021	0.66451	0.49200	0.0586*	
H3	0.14255	1.13832	0.30825	0.0742*	
H5	0.07821	0.70656	0.45048	0.0694*	
H7A	0.18460	0.43399	0.50812	0.1034*	
H7B	0.30533	0.49271	0.57044	0.1034*	
H7C	0.25797	0.39442	0.45505	0.1034*	
H8A	0.31727	1.17980	0.33141	0.1162*	
H8B	0.32495	1.00412	0.26651	0.1162*	
H8C	0.40502	1.02536	0.39246	0.1162*	
H9A	−0.05817	1.03567	0.27711	0.1608*	
H9B	0.00533	1.16026	0.38250	0.1608*	
H9C	−0.04105	0.96399	0.39150	0.1608*	
H11	0.34159	0.43378	0.09636	0.0626*	
H12	0.50176	0.34047	0.25128	0.0599*	

### Atomic displacement parameters (Å^2^)

**Table:**

	*U*^11^	*U*^22^	*U*^33^	*U*^12^	*U*^13^	*U*^23^
S1	0.0449 (3)	0.0634 (3)	0.0357 (2)	0.0060 (2)	0.0122 (2)	0.0011 (2)
N1	0.0442 (7)	0.0644 (9)	0.0332 (6)	0.0155 (7)	0.0148 (5)	0.0007 (6)
N2	0.0449 (7)	0.0542 (8)	0.0398 (7)	0.0100 (6)	0.0217 (6)	0.0047 (6)
C1	0.0410 (8)	0.0480 (9)	0.0323 (7)	0.0071 (7)	0.0147 (6)	−0.0023 (6)
C2	0.0594 (10)	0.0491 (9)	0.0441 (9)	0.0067 (8)	0.0263 (8)	0.0009 (7)
C3	0.0747 (13)	0.0597 (11)	0.0479 (10)	0.0267 (10)	0.0268 (9)	0.0082 (8)
C4	0.0539 (10)	0.0921 (15)	0.0403 (9)	0.0287 (10)	0.0182 (8)	0.0018 (9)
C5	0.0448 (9)	0.0857 (14)	0.0432 (9)	0.0020 (9)	0.0212 (7)	−0.0026 (9)
C6	0.0481 (9)	0.0532 (9)	0.0347 (7)	0.0004 (7)	0.0174 (7)	−0.0039 (7)
C7	0.0830 (14)	0.0585 (12)	0.0719 (13)	−0.0024 (10)	0.0425 (12)	0.0080 (10)
C8	0.0980 (17)	0.0590 (12)	0.0915 (17)	−0.0005 (12)	0.0585 (15)	0.0086 (11)
C9	0.0779 (16)	0.166 (3)	0.0768 (16)	0.0672 (19)	0.0362 (13)	0.0210 (17)
C10	0.0377 (7)	0.0420 (8)	0.0340 (7)	0.0021 (6)	0.0158 (6)	0.0044 (6)
C11	0.0656 (11)	0.0535 (10)	0.0407 (8)	−0.0042 (8)	0.0282 (8)	−0.0051 (7)
C12	0.0594 (10)	0.0505 (10)	0.0500 (9)	0.0078 (8)	0.0344 (8)	0.0015 (7)

### Geometric parameters (Å, º)

**Table:**

S1—C10	1.7428 (15)	C6—C7	1.509 (3)
S1—C11	1.720 (2)	C11—C12	1.335 (3)
N1—C1	1.421 (2)	C3—H3	0.9300
N1—C10	1.354 (2)	C5—H5	0.9300
N2—C10	1.305 (2)	C7—H7A	0.9600
N2—C12	1.380 (2)	C7—H7B	0.9600
N1—H1	0.8600	C7—H7C	0.9600
C1—C6	1.393 (3)	C8—H8A	0.9600
C1—C2	1.401 (2)	C8—H8B	0.9600
C2—C3	1.388 (3)	C8—H8C	0.9600
C2—C8	1.505 (4)	C9—H9A	0.9600
C3—C4	1.379 (3)	C9—H9B	0.9600
C4—C5	1.383 (3)	C9—H9C	0.9600
C4—C9	1.511 (4)	C11—H11	0.9300
C5—C6	1.384 (3)	C12—H12	0.9300
			
C10—S1—C11	88.94 (8)	C4—C3—H3	119.00
C1—N1—C10	122.23 (14)	C4—C5—H5	119.00
C10—N2—C12	110.02 (15)	C6—C5—H5	119.00
C1—N1—H1	119.00	C6—C7—H7A	109.00
C10—N1—H1	119.00	C6—C7—H7B	109.00
N1—C1—C2	119.41 (17)	C6—C7—H7C	109.00
N1—C1—C6	119.75 (13)	H7A—C7—H7B	109.00
C2—C1—C6	120.82 (17)	H7A—C7—H7C	109.00
C1—C2—C3	117.6 (2)	H7B—C7—H7C	109.00
C3—C2—C8	120.31 (19)	C2—C8—H8A	109.00
C1—C2—C8	122.1 (2)	C2—C8—H8B	109.00
C2—C3—C4	122.9 (2)	C2—C8—H8C	109.00
C3—C4—C9	121.2 (2)	H8A—C8—H8B	109.00
C3—C4—C5	117.7 (2)	H8A—C8—H8C	109.00
C5—C4—C9	121.1 (2)	H8B—C8—H8C	110.00
C4—C5—C6	122.1 (2)	C4—C9—H9A	110.00
C1—C6—C5	118.75 (15)	C4—C9—H9B	110.00
C5—C6—C7	120.70 (19)	C4—C9—H9C	109.00
C1—C6—C7	120.55 (18)	H9A—C9—H9B	110.00
S1—C10—N2	114.35 (12)	H9A—C9—H9C	109.00
S1—C10—N1	121.78 (13)	H9B—C9—H9C	109.00
N1—C10—N2	123.87 (14)	S1—C11—H11	125.00
S1—C11—C12	110.05 (14)	C12—C11—H11	125.00
N2—C12—C11	116.6 (2)	N2—C12—H12	122.00
C2—C3—H3	119.00	C11—C12—H12	122.00
			
C11—S1—C10—N1	−179.66 (15)	N1—C1—C6—C5	−179.71 (14)
C11—S1—C10—N2	−0.05 (14)	N1—C1—C6—C7	0.6 (2)
C10—S1—C11—C12	0.38 (17)	C2—C1—C6—C5	2.0 (2)
C10—N1—C1—C2	−76.7 (2)	C2—C1—C6—C7	−177.69 (16)
C10—N1—C1—C6	104.96 (18)	C1—C2—C3—C4	3.6 (3)
C1—N1—C10—S1	6.9 (2)	C8—C2—C3—C4	−174.12 (19)
C1—N1—C10—N2	−172.72 (16)	C2—C3—C4—C5	−0.8 (3)
C12—N2—C10—S1	−0.29 (19)	C2—C3—C4—C9	179.1 (2)
C12—N2—C10—N1	179.31 (17)	C3—C4—C5—C6	−1.7 (3)
C10—N2—C12—C11	0.6 (3)	C9—C4—C5—C6	178.44 (18)
N1—C1—C2—C3	177.48 (15)	C4—C5—C6—C1	1.1 (3)
N1—C1—C2—C8	−4.8 (2)	C4—C5—C6—C7	−179.29 (18)
C6—C1—C2—C3	−4.2 (2)	S1—C11—C12—N2	−0.7 (3)
C6—C1—C2—C8	173.51 (17)		

### Hydrogen-bond geometry (Å, º)

**Table:**

*D*—H···*A*	*D*—H	H···*A*	*D*···*A*	*D*—H···*A*
N1—H1···N2^i^	0.86	2.16	2.944 (2)	151

Symmetry code: (i) −*x*+1, −*y*+1, −*z*+1.
